# Bridging the gap: enhancing data science and leadership knowledge and skills in the context of the public health workforce

**DOI:** 10.3389/fpubh.2025.1505869

**Published:** 2025-04-29

**Authors:** Diana Hamer, Madelyn Gustafson, Christine Ortiz Gumina, Danielle C. Landis, Patricia Bockelman, Elaina Perry, Sarah D. Matthews

**Affiliations:** ^1^National Network of Public Health Institutes, New Orleans, LA, United States; ^2^Health Communication Consultants, Inc., Winter Garden, FL, United States

**Keywords:** public health data science, health leadership, workforce development, public health, public health education

## Abstract

**Introduction:**

This study addresses critical gaps in public health workforce development, focusing on data science and leadership skills amidst increasing data accessibility and complexity in public health practice.

**Methods:**

Quantitative and qualitative research methods were employed, including listening sessions with diverse public health professionals. Recruitment and post-session surveys were conducted, and session transcripts were analyzed using phenomenological and categorical coding methods based on the PPP Framework.

**Results:**

The research identified three core concepts in public health data science and leadership: data science, data literacy, and data-informed leadership. Clear definitions for each concept were developed. Significant gaps in workforce capacity, accessibility, and training were highlighted, particularly in aligning academic curricula with real-world public health needs.

**Analysis:**

The study revealed a disconnect between current public health programs and workforce preparedness, performance, and practice. Participants emphasized the need for comprehensive leadership development programs and integration of data science and leadership components into existing curricula.

**Discussion:**

This work provides a foundation for strengthening the public health workforce by identifying key concepts and gaps in data science and leadership. The findings have implications for policy development, resource allocation, and building a competent workforce capable of leveraging data for improved public health outcomes. This research contributes to advancing the field of public health by communicating scientific knowledge crucial for future breakthroughs in workforce development.

## 1 Introduction

### 1.1 Challenges in public health data

Data constitutes a critical resource in the domain of public health, underpinning evidence-based practices, intervention strategies, and the enhancement of community health and safety. Public Health data is used to understand the burden of disease, predict and improve health outcomes, recognize solutions to disparities, and provide evidence of the impact of interventions (1). Since its formal establishment as a field, public health has leveraged data to fulfill its mission to enhance overall health and wellbeing for all people. This mission was highlighted in the 1988 Institute of Medicine (IOM) report, *The Future of Public Health* (2). The updated 2002 IOM report’s call to action was to strengthen governmental public health through advancements in their capabilities in surveillance (3). To achieve this, data collection and analysis required modernization, the pursuit of which developed the specialty of Public Health Informatics and Data Science. Over the past two decades, with greater attention on public health data, surveillance, collection and application for decision-making, the role and vulnerabilities of data science have become increasingly apparent. During the inaugural Data Science in Public Health Summit, participants described data science as an overarching term for four emerging trends: increased data availability and complexity, development of computational methods, advances in computational infrastructure, and growing concern around scientific rigor.

### 1.2 Challenges in the public health workforce

The COVID-19 pandemic underscored numerous deficiencies in public health systems, including inadequate workforce capacity exacerbated by years of underfunding, as highlighted by a reduction of 40,000 public health jobs between the 2008 Great Recession and the pandemic’s onset (4, 5). The lack of support for national public health efforts negatively impacts efforts to effectively deliver the Foundational Public Health Services, described as the bare minimum communities should be offered.

### 1.3 Examining intersecting gaps between the public health workforce and public health data science

*The public health workforce interests and needs survey (PH WINS)* has brought forth gaps within the field (6) including training needs in strategic thinking, diversity, equity, and inclusion (DEI), data decision-making, and policy engagement. As Fraser et al. (7) pointed out, PH WINS is not only a dataset but an advancement that can promote data-driven workforce planning in governmental public health. PH WINS reveals that one of the biggest gaps in public health is the lack of multisector and crosscutting leadership and the need to transition from individual to collective-focused leadership. The current public health workforce is further challenged by the widespread availability of big data and user-generated data that requires new analytical methods and data science developments to guide public health practice and policy. Data science plays an essential role in informing public health decision making, however inadequate investments in the workforce and infrastructure, including those related to data science leadership development, present challenges to the workforce’s ability to keep pace with the dynamic and rapidly evolving landscape of data availability (8). Inadequate systems in surveillance, information technology, and data processing prevent time-appropriate interventions centered in scientifically sourced data and free of misinformation to be provided to the public (5).

### 1.4 The role of academics in addressing gaps in the public health data science workforce

The new shifts and advances in data science and public health leadership are reflected in current trends of academic offerings. Data science undergraduate, graduate, and upskilling programs are increasing in popularity (1). The increasing accessibility of data and the need for collection, analysis, and visualization highlight the disconnect between the public health workforce knowledge and needs (8), pressuring academic and training institutions to address how to adequately prepare students. However, there is little information on the employment outcomes of recent public health graduates or knowledge and skills matching between their education and the core public health workforce (5). The absence of this data makes it challenging for the governmental public health workforce to understand recent graduates’ data science and leadership skills. A better understanding of the misalignment between academic training and the workforce needs is required to establish a sustainable, competent pipeline.

Mirin et al. (9) reviewed educational programs in public health data science across top domestic and global public health schools, finding that most data science degrees are awarded at the master’s level, primarily within epidemiology, biostatistics, and health informatics. They noted a significant gap in access to data-intensive courses for students in socio-behavioral fields, suggesting a need for further research to align public health data science curricula with the technical and essential skills required for successful careers in data science and leadership (9). This is exemplified in a recent study that identified data science and leadership as two of the four domains of skills required by local public health departments for responding to community needs and improving health outcomes (10).

Through a commitment to advancing the public health workforce to meet community needs, the National Network for Public Health Institutes (NNPHI) began exploring the landscape of data science and leadership in public health. NNPHI aims to address both the internal needs of the workforce and their ability to meet the evolving demands of the communities they serve. In partnership with the Centers for Disease Control and Prevention’s Public Health Leadership and Education, Advancing Health Equity and Data Science (Public Health LEADS) program, NNPHI collaborated with Health Communications Consultants, Inc. to focus on the data science and leadership skills across current public health career pathways for mid to senior-level professionals (11).

This exploration served as a foundational effort to enhance the public health workforce. Through listening sessions, we identified and defined key concepts in public health data science and leadership, as well as gaps in capacity, accessibility, education, and training. This paper discusses these concepts and methods, synthesizing participant insights into three core areas: data science, data literacy, and data-informed leadership.

## 2 Materials and methods

An observational, cross-sectional evaluation was conducted in July 2023. The data collection methods included three tools: (1) recruitment survey (Table 1), (2) listening sessions, and (3) post listening session survey (Table 2).

**Table 1:** Recruitment survey.

Recruitment survey-public health leads				
Question number	Survey section	Survey logic	Type of question	Question
1	Introduction	None	Informational	Thank you for your interest in participating in our listening sessions! By continuing in the survey, you acknowledge that your participation is voluntary. Your responses will be kept confidential and will be used to determine your eligibility for the listening sessions. You may choose to terminate your participation at any time and can skip any question you choose. By submitting this survey, you consent to participation and affirm you are 18 years or older. If you have any questions about this evaluation, please contact Sarah Matthews, PhD, via email at sarah.matthews@healthcommunicationsconsultants.com. The purpose of this listening session is to understand the current governmental public health workforce perception of public health data science and public health leadership and understand gaps in workforce capacity, training, and education. The information learned in this listening session will help to: 1. Define key public health data science and leadership concepts for the workforce, 2. identify current gaps in the data science and leadership needs of the current workforce, 3. identify current gaps in data science and leadership capacity, accessibility, training, and education needs of the public health workforce, 4. address ways that systems undermine efforts to create a more diverse public health workforce and 5. align workforce needs and current public health programs with data science and leadership curricula.
	Introduction	None	Informational	This project is supported by the Centers for Disease Control and Prevention of the U.S. Department of Health and Human Services (HHS) as part of a financial assistance award (NU36OE000016–01-00, titled Strengthening Environmental Health—Building Capacity for a More Diverse and Representative Workforce) totaling $366,000 with 100 percent funded by CDC/HHS. The contents are those of the author(s) and do not necessarily represent the official views of, nor an endorsement, by CDC/HHS, or the U.S. Government.
2	Survey	None	Open text	Please provide the following information: Name Organization Name (Please do not use abbreviations) County State Email Phone number Certifications, Credentials
3	Survey	Skip logic to 3a if choices c-h, Skip to 4 if a-b	Multiple select	What degrees have attained, select all that apply. a. Some high school b. High school degree or equivalent (e.g., GED) c. High school technical, please specify (Text box) d. Some college but no degree e. Associate degree or certificate f. Bachelor’s degree g. Master’s degree h. Doctoral degree (MD, DO, PhD, DDS, JD, etc.) i. Other (please specify): __
3a	Survey	Destination from 3.	Multiple select	What are the subject of your degree(s) in? After your selection please write which degree the subject applies in the text box. a. Business (Text box) b. Communications (Text box) c. Dental (Text box) d. Education (Text box) e. Engineering (Text box) f. Environmental Health (Text box) g. Health Services/Administration (Text box) h. Hospitality (Text box) i. Human Resources (Text box) j. Laboratory Science (Text box) k. Liberal Arts/Humanities (please specify) (Text box) l. Mathematics/Economics (Text box) m. Medicine (Text box) n. Nursing (Text box) o. Nutrition (Text box) p. Occupational Health and Industrial Hygiene (Text box) q. Public Health (Text box) r. Science (please specify) (Text box) s. Social Work (Text box) t. Technology (Text box) u. Other (please specify) (Text box)
4	Survey	Destination from 3.	Multiple select	How would you best describe your Race/Ethnicity (select all that apply) a. Asian b. American Indian or Alaskan Native c. Black/African American d. Native Hawaiian or other Pacific Islander e. Hispanic, Latino or Spanish origin of any race f. Non-Hispanic g. White h. Two or more i. Some other race (please specify) j. Decline to state
5	Survey	None	Text space limited	What is your age?
6	Survey	None	Single select	How do you best identify in terms of gender? a. Male b. Female c. Non-Binary d. Prefer not to say e. Other (please specify) (Text Box)
7	Survey	Branch for “Yes” to 7 a-c to 8. Branch for “No” Response to 7 f to 22.	Multiple select	Do you work or have experience in public health leadership, public health workforce development, data science education, public health recruitment and retention and/or public health leadership curricula. Select all that apply. a. Public health leadership b. Public health workforce development c. Public health programs with data science d. Public health recruitment and retention e. Public health leadership curricula f. No, I do not have experience in any of these areas
8	Survey	None	Single select	Approximately how much time have you worked in public health leadership, public health workforce development, data science education, public health recruitment and retention and/or public health leadership curricula? a. Less than 6 months b. 6 months to 1 year c. 2–5 years d. 6–10 years e. 11–15 years f. Greater than 15 years
9	Survey	Branch for “Yes” to 9–9a. Branch “No” to 9–10.	Single select	During your years of experience in public health leadership, public health workforce development, data science education, public health recruitment and retention and/or public health leadership curricula did you supervise or manage others? a. Yes b. No
9a	Survey	Destination from 9.	Single select	During your years of experience in public health leadership, public health workforce development, data science education, public health recruitment and retention and/or public health leadership curricula how long did you supervisor or manage others? a. Less than 6 months b. 6 months to 1 year c. 2 years to 5 years d. 6 years to 10 years e. 11 years to 15 years f. Greater than 15 years
10	Survey	Destination from 9.	Single select	Which best describes the work setting in which you currently work (If retired, please indicate which best describes the work setting in which you most recently worked prior to retirement) in public health leadership, public health workforce development, data science education, public health recruitment and retention and/or public health leadership curricula. a. Local health agency b. State health agency c. Territorial health agency d. Federal health agency e. Tribal health agency f. Educational/academic institution g. Private nonprofit organization h. Private foundation i. Personal health service industry (Hospital, Rehabilitation Center, Assisted Living Facility, Dental Facility, Pharmacy, Outpatient facility, Physicians Office) j. Other (Please specify) (Text Box)
11	Survey	None	Single select	Which best describes the employer(s) in which you developed most of your experience in public health leadership, public health workforce development, data science education, public health recruitment and retention and/or public health leadership curricula. a. Local health agency b. State health agency c. Territorial health agency d. Federal health agency e. Tribal health agency f. Educational/academic institution g. Private nonprofit organization h. Private foundation i. Personal health service industry (Hospital, Rehabilitation Center, Assisted Living Facility, Dental Facility, Pharmacy, Outpatient facility, Physicians Office) j. Other (Text Box)
12	Survey	None	Single select	Which best describes the general area where you work? a. Urban b. Suburban c. Rural d. Tribal area e. Other (please specify) (Text Box)
13	Survey	None	Single select	Which best describes your occupation in which you developed experience in public health leadership, public health workforce development, data science education, public health recruitment and retention and/or public health leadership curricula? a. Academia- Curriculum developers, Professors/Faculty, Coordinators/Staff b. Academia- Current students from public health programs c. State, local, tribal, and territorial health departments, and federal agencies- Programmatic Staff d. State, local, tribal, and territorial health departments, and federal agencies- Directors & Managers e. State, local, tribal, and territorial health departments, and federal agencies- Administrative staff f. State, local, tribal, and territorial health departments, and federal agencies- Recent graduates g. National public health non-profits and other related organizations—Programmatic Staff h. National public health non-profits and other related organizations—Directors & Managers i. National public health non-profits and other related organizations –Administrative staff. j. National public health non-profits and other related organizations –Recent Graduates k. Private-for-profits and other related organizations-Programmatic Staff l. Private-for-profits and other related organizations- Direct and Managers m. Private-for-profits and other related organizations- Recent graduates n. Other (e.g., volunteer, intern) (Text box please describe)
14	Survey	None	Open text	How would you describe the strengths of the current governmental public health workforce in terms of (a) public health data science and/or (b) public health leadership?
15	Survey	None	Open text	In your opinion, what are the current gaps in governmental public health workforce capacity as it relates to (a) public health data science and/or (b) public health leadership?
16	Survey	None	Open text	In your opinion, what are the current gaps in governmental public health workforce training and education as it relates to (a) public health data science and/or (b) public health leadership?
17	Survey	None	Open text	What are the most pressing governmental public health workforce needs in (a) public health data science and (b) public health leadership?
18	Survey	None	Open text	What are the current barriers or challenges in the governmental public health workforce’s needs for (a) public health data science and/or public health leadership?
19	Survey	None	Open text	What are some innovative or best practice ideas to address the barriers/challenges for the governmental public health workforce needs in (a) public health data science and/or (b) public health leadership?
20	Survey	Branch “Yes” to 20–20a. Branch “No” to 20–21.	Single select	I am willing to participate in a virtual focus group to provide information about public health leadership, public health workforce development, data science education, public health recruitment and retention and/or public health leadership curricula. a. Yes, I am willing to participate in a virtual focus group. b. No, I am not willing to participate in a virtual focus group.
20a	Survey	Destination from 20	Multiple select	As we work on the scheduling of the group Listening Session (Virtual Focus Group) which of these Date and Time would work with your schedule? Please select your top 2 choices. a. Wednesday, July 12, 2023, from 1:00 PM-3:00 PM EST (10:00 AM-12:00 PM PST) b. Thursday, July 13, 2023, from 12:00 PM-2:00 PM EST (9:00 AM-11:00 AM PST) c. Friday, July 14, 2023, from 12:00 PM-2:00 PM EST (9:00 AM-11:00 AM PST) d. Wednesday, July 19, 2023, from 12:00 PM-2:00 PM EST (9:00 AM-11:00 AM PST)
21	Survey	Destination from 20	Open text	Do you have any additional thoughts or comments to share?
22	Survey	Destination from 7	Informational	At this time, you do not meet the recruitment requirements for this project. We thank you for your time.
	End block	None	Informational	We thank you for your time spent taking this survey. Your responses have been recorded and we will be in contact with you via email within x days regarding your participation in the focus group session. Please be sure to check your spam folder or add us to your safe sender list.

**Table 2:** Post listening session survey.

Question number	Survey section	Survey logic	Type of question	Question
1	Introduction	None	Informational	Thank you for participating in the listening session for understanding the current governmental public health workforce conceptualization of public health data science and public health leadership and understanding gaps in workforce capacity, training, and education. Please complete this closeout survey for our evaluation and the opportunity to be selected as one (1) of forty (40) eligible participants to receive their choice of either a 1-year subscriptions to AI services/training (e.g., OpenAi, ChatGPT4, ChatSonic Premium) or leadership-data training (e.g., LinkedIn training) for a section of your staff valued at $240. If you have any questions about this evaluation, please contact Sarah Matthews, PhD, via email at sarah.matthews@healthcommunicationsconsultants.com
2	Survey	None	Open text	Please provide the following information. a. Name b. Organization c. State d. Unique Identifier
3	Survey	None	Single select	In which Listening Session did you participate? a. Wednesday, July 12, 2023, from 1:00 PM-3:00 PM EST (10:00 AM–12:00 PM PST) b. Thursday, July 13, 2023, from 12:00 PM-2:00 PM EST (9:00 AM–11:00 AM PST) c. Friday, July 14, 2023, from 12:00 PM-2:00 PM EST (9:00 AM–11:00 AM PST) d. Wednesday, July 19, 2023, from 12:00 PM-2:00 PM EST (9:00 AM–11:00 AM PST)
4	Survey	None	Open text	Reflecting on your listening session conversation, do you have any additional information or clarifications to share?
5	Survey	None	Multiple select	5. What was your motivation for participating in the listening session? (Select all that apply) a. Willingness to help; Provide better support in the field and community (Altruistic Motivation). b. Interesting evaluation; Curiosity (Intellectual Motivation). c. Incentive offered. d. Opportunity to learn from others. Individual professional development e. The request to participate came from a peer or someone I respect. f. Camaraderie or to have a shared experience. g. Other (please specify) (Text box)
6	Survey	None	Open text	How would you describe the current governmental public health workforce in terms of (a) public health data science and/or (b) public health leadership
7	Survey	None	Open text	How would you describe the strengths of the current governmental public health workforce in terms of (a) public health data science and/or (b) public health leadership?
8	Survey	None	Open text	In your opinion, what are the current gaps in governmental public health workforce capacity as it relates to (a) public health data science and/or (b) public health leadership?
9	Survey	None	Open text	In your opinion, what are the current gaps in governmental public health workforce training and education as it relates to (a) public health data science and/or (b) public health leadership?
10	Survey	None	Open text	What are the most pressing governmental public health workforce needs in (a) public health data science and/or (b) public health leadership?
11	Survey	None	Open text	What are the current barriers or challenges in the governmental public health workforce’s needs for (a) public health data science and/or (b) public health leadership?
12	Survey	None	Open text	What are some innovative or best practice ideas to address the barriers/challenges for the governmental public health workforce needs in (a) public health data science and/or (b) public health leadership?
13	Survey	None	Single select	13. Would you be willing to participate in similar work in the future with the National Networks of Public Health Institutes (NNPHI)? a. Yes b. No c. Maybe
14	Survey	None	Open text	Do you have any additional thoughts or comments to share?
15	Survey	None	Single select	Select one of the following statements. a. Yes, I want to be entered for the opportunity to be selected as one (1) of forty (40) eligible participants to receive their choice of either a 1-year subscriptions to AI services/training (e.g., OpenAi, ChatGPT4, ChatSonic Premium) or leadership-data training (e.g., LinkedIn training) for a section of your staff valued at $240. b. No, I do not want to be entered for a chance to receive the participation incentive.

### 2.1 Measurement tools

The recruitment survey was comprised of 21 questions: 13 multiple choice questions, seven open-ended, and one text-limited. Utilizing Qualtrics, the recruitment survey was administered to a convenience sample recruited via social media and personal connection outreach from June 11, 2023, through July 11, 2023.

A semi-structured conversational tool (Table 3) was created to help guide the listening sessions. Listening sessions were recorded and transcribed for use.

**Table 3:** Semi-structured conversational tool.

Listening session conversation tool		
Conversation segment	Interviewer prompt question	Notes
Protocol reminders	Protocol reminders: Make note if a participant drops. Assign participant numbers. During conversation, encourage multiple people to answer the same question, but with their unique stories; the goal is to find similarities and contrasts. With each story, make note of phrases that indicate: Emotion (e.g., “I felt frustrated.” or “We were so happy that worked out!”). Follow up on emotive statements with clarifying what happened after the event that triggered that emotion (was it sustained or replaced with a different event). Process (e.g., “It was easy because…” or “We have a requirement to do XYZ”). Clarify how they came to learn that process. Context complexity (e.g., “We could not do X because Y” or “We were told to do A but that never works because of B”). Ask about contingency plans and how they have been prepared for unanticipated challenges.	
Welcome	We want to start with thanking you for participation.	*This should be delivered conversationally, to help participants feel comfortable. It does not need to be read verbatim.*
	Please remember that your participation is entirely voluntary.	*Portions that refer to informed consent details may be abbreviated, with a gentle reminder to review the consent and contact information, with an invitation to ask any questions.*
	In the invitation, you were assigned a participant identification number. Please use this number as your identifier in the name field on Zoom (Allow participants to rename themselves, then start the recording).	
	We are recording these listening sessions. If you do not wish to be recorded, please leave the zoom platform now. If you are not actively speaking or preparing to speak, please keep muted.	
	Keep the background noise to a minimum when you are unmuted.	
	Speak clearly into the microphone on your computer or on the phone line.	
	Refrain from shuffling papers, typing loudly, or talking among each other.	
	Please take a moment and check where you placed your microphone. If you are in a room with other people sharing the same dial-in, place the microphone near the participants who are talking.	
	If you have an external microphone this might be a better option than a built-in one for better sound quality.	
	Please take a moment and accurately put your zoom name into the name section. The transcripts for these recording sessions will capture that name when you are speaking.	
	Contributions added in the Chat Box function will be repeated out loud in order to ensure that they are captured by the transcript and to allow all participants and the facilitator to consider new perspectives/	
	You have been invited to participate in this listening session hosted by the National Network of Public Health Institutes (NNPHI) and under the research direction of Health Communications Consultants, Inc.	
	The purpose of this listening session is to understand the current governmental public health workforce perception of public health data science and public health leadership and understand gaps in workforce capacity, training and education.	
	The information learned in this listening session will help to (1) define key public health data science and leadership concepts for the workforce (2) identify current gaps in the data science and leadership needs of the current workforce (3) identify current gaps in data science and leadership capacity, accessibility, training, and education needs of the public health workforce, (4) address ways that systems undermine efforts to create a more diverse public health workforce and (5) align workforce needs and current public health programs with data science and leadership curricula.	
	This listening session is one of four sessions. A facilitator will be posing questions to you for discussion. The listening sessions will be recorded, a written transcript will be produced and there are note-takes present on the zoom platform. Your responses will remain confidential, and no names will be included in the final external report. Participants should not record the listening sessions.	
	You can choose whether or not to participate in the listening session and you may stop at any time during the course of the session. Please note that there are no right or wrong answers to the posed questions. We want to hear the many varying viewpoints and would like for everyone to contribute their thoughts. Please feel free to be honest even when your responses counter those of other group members.	
	Your participation benefits the public health workforce by improving the ability to meet the public health workforce’s needs. No risks are anticipated beyond those experienced during an average conversation.	
	Should you choose to participate, you are asked to respect the privacy of other listening session group members by not disclosing any content discussed during the session. Health Communications Consultants, Inc. will analyze the data and your responses will remain confidential.	
	If you have any questions or concerns about the listening sessions, please contact Dr. Sarah Matthews at sarah.matthews@healthcommunicationsconsultants.com.	
	Does anyone have any questions about the listening session before we begin?	
	*Answer any questions.*	
	One more reminder before we begin: Your participation is entirely voluntary. There is no penalty for dropping at any time.	
	At this time, by continuing to be logged on to the Zoom platform, you indicate that you understand the information presented and agree to participate fully under the conditions stated above.	
Conversation	OK, we’d like to start the listening session by explaining a bit about the process for this conversation. Our priority today is to listen to you tell your stories.	The general pattern for these questions is: (1) “tell me about a specific time when you _____.” (2) listen for examples of skills and follow up with “tell me more about _____.” (3) listen for indicators of sub-skills and related skills, gaps, trends, etc. (4) ask for any similar experiences. (5) ask for different/contrasting experiences.
	This means, I do not want to assume that I know what is in your head, so I will frequently be asking you to clarify things that may feel pretty obvious to you. So, do not be surprised when you hear me say something like, “what do you mean by ____(and use your own phrase)?” or “Could you describe what that was like?”	Then, we can loop through these questions with similar phrasings but focusing on variations, such as by context, access to resources, organizational differences, and outcomes.
	*If there are terms to define that will be used throughout the conversation, now is the time to do so.*	While there may be some questions that are speculative (such as asking what skills would be helpful), most of the questions must be focused on what *has worked or has failed to work,* so that it can be grounded in experience.
	So, let us begin with thinking about your recent use of data in your public health work.	
		*Public health data science definition and definition of data science and leadership are of interest.
	Could anybody describe an experience using data on the job?	
	*Note, allow for pauses and for participants to take time…especially with these icebreaking conversations.*	
	*if no one answers the initial question, ask if anyone has received valuable training.	
	*Once someone gives that answer, follow on with questions:*	
	Does anyone have similar experiences?	
	Can anyone describe an experience where there was a different point of view between supervisor/manager/leader and the team in regard to data use?	
	Let us talk about how data science impacts the public facing aspects of your job.	
	Can anyone provide an example of training you have received for communicating data to diverse groups?	
	Would anyone be willing to share a story about a time you have worked to share data insights with a group, but the efforts were unsuccessful?	
	Can anyone describe how leaders support a team’s use of data?	
	Can anyone describe an experience where a lack of data skills on a team impacted outcomes?	
	Can anyone describe an experience where lack of leadership skills on a team impacted outcomes?	
	Can anyone tell me about a time when a lack of leadership skills interfered with meeting outcomes?	
	Can anyone describe a time when you did not have the skills needed for the work being asked? (i.e., when they were a novice or new to their job in public health)	

The post listening session survey was comprised of 14 questions: 4 multiple choices, 9 open-ended and 1 demographics-related question. Utilizing Qualtrics, the post listening session survey was administered from July 12, 2023, through July 27, 2023.

### 2.2 Participants and procedure

#### 2.2.1 Recruitment

The recruitment survey used the online Qualtrics platform, targeting members of the public health workforce from academia, government health departments, public health nonprofits, and related organizations. Convenience and snowball sampling methods were used through the research team’s networks to expand the participant pool. A recruitment email and flyers were sent to the participant pool with consent to participate assessed by their continuation through the recruitment process. Follow-up email communications were sent to individuals who responded to the initial contact. Participants were given the option of selecting one of four 90-min listening sessions in which to participate. A review of recruitment survey responses was done to ensure that potential supervisory and subordinate coworkers were not in the same sessions. Once a session selection was made, an email invitation with a unique identifier was sent to the participant. A reminder email was sent 1 day prior to the scheduled date to improve the participation rate and ensure timely attendance. Persons unable to make their initial selection were communicated with to reschedule for their second selection or for a later listening session date.

#### 2.2.2 Listening sessions

We applied phenomenological methods of qualitative research for the listening sessions to encourage open conversation on the topics to be explored. Phenomenological methods are used for the purpose of understanding the nature of the experiences while avoiding interviewers inducing speculation (e.g., “*Why do you think your supervisor wanted you to do that?*” or “*How would you feel if you had received a different kind of training?*”) (12–14). The approach allows for naturalistic conversations that flow with the participants’ responses as opposed to traditional focus group methods which utilize the same questions for each focus group and can bias participants’ responses in the direction of the specific question. It encourages participants to share their stories and lived experiences in a safe and supportive context while the conversation flow provides insights into the priorities of specific decisions and their consequences. The approach also provides benefits for participants by providing opportunities to be authentically “seen and heard” (13, 14). A semi-structured conversational tool was created to help guide the sessions (Table 3). Prior to entering the Zoom platform, participants were renamed using the unique identifier assigned to them to ensure anonymity in the evaluation processes. Participants were encouraged to turn their cameras on during the session, but it was not required. A PowerPoint slide deck with welcome and thank you slides containing access to the post listening session survey were created to initiate and end the conversation. The survey link was provided to participants at the conclusion of the listening session and provided again prior to the closure of the survey. To be eligible for the participant incentive, participants needed to complete the post listening session survey which was also hosted on a Qualtrics platform. Listening sessions were recorded and transcribed with a transcription service. The evaluation team observed and took notes during each session. The MPHI (Michigan Public Health Institute) Institutional Review Board determined all research methods presented were exempt from further review.

### 2.3 Analysis

Descriptive analysis was conducted on the recruitment survey and post listening survey data results. Open-ended questions in both surveys specified that participants’ answers addressed two factors: (a) public health data science, and (b) public health leadership. The resulting binary code used in coding and analysis could be interpreted as discrete or unified parts.

After the first coding phase, categorical coding was used on the listening session transcripts by three independent coders to break the data down further into three discrete parts: (1) People, (2) Process, and (3) Products (The “PPP Framework”). The framework is one frequently used in organizational improvement. For our analysis, the PPP Framework parts were described as follows:

- People: people within the public health workforce and how they engage with one another.
- Process: processes and procedures within public health organizations.
- Products: products or services offered, provided, or needed by the public health organizations.
- Environment: external or internal environmental factors that affect the PPP Framework (e.g., political climate, social variables, workforce culture, etc.).

The exploratory nature of the evaluation warranted an inductive approach. Open coding was conducted to create additional themes/sub-categories. Codes were aggregated and condensed across coders per listening session and then across all listening sessions. There were eight axial categories:

- *Collaboration*: activities of two or more people or organizations working toward a common goal, which could include products, processes, desired effects, outcomes, etc.
- *Funding/Resources*: finances, materials, staff, or other assets needed to function effectively.
- *Recruitment and retention*: the process of identifying, attracting, interviewing, selecting, and hiring people to join a public health organization and then keeping those employees within the agency and/or in the public health workforce.
- *Systems improvemen*t: activities to make the public health system or related public health processes, products, or people more effective, efficient, transparent, fair, inclusive, etc. (e.g., improvements in data modernization, policy, development, etc.).
- *Tools/technology*: devices, software, hardware, applications, programming, programming languages, information technology (IT) systems.
- *Workforce*: people within the public health workforce and their work-related attributes.
- *Workforce development*: processes and products that educate and train individuals and groups to effectively meet the current and future needs of the agencies involved in planning for and delivering public health services.
- *Other*: includes responses that fell outside of the eight axial codes (e.g., factors such as awareness, value, time, etc.).

## 3 Results

### 3.1 Participant profile

There were 267 unique email invitations sent to persons identified in the public health workforce during the recruitment timeframe resulting in a 25.8% response rate (69/267). Sixty-five (65) respondent surveys were retained for analysis; of which twenty-six (26) respondents participated in a listening session (LS). The average age of respondents was 40 years with a range of 22–64 years of age, while the age of listening session participants was an average of 43 years and range of 31–63 years of age. Respondents and listening session participants were majority white [Survey: 55% (36); LS 53% (21)] and female [Survey: 70% (46); LS 54% (14)]. Respondents and listening session participants are highly educated with bachelor’s degrees [Survey: (36); LS (1)], masters’ degree [Survey: 74% (48): LS 50% (13)] and doctoral degrees [Survey: 28% (18); LS: 46% (12)]. Forty-nine percent of respondents identified that their advanced degree was in the field of public health (*n* = 32). Respondents identified their experience in public health programs with data science (*n* = 55), public health leadership (*n* = 41), public health workforce development (*n* = 38), public health recruitment and retention (*n* = 24), and public health leadership curricula (*n* = 14) as >2 years of experience (*n* = 59). Their current work environment included local health agency (*n* = 16), state health agency (14), educational/academic institute (12), private nonprofit organization (10), private for profit (5), federal health agency (2) and other (5) which they classified as urban (30), suburban (18), Other (12, mixed settings), rural (3) and tribal area (1). See Supplementary Table 1 for additional information on participant profiles.

#### 3.1.1 Motivation for participation

Of the 26 listening session participants, 14 responded to the post listening session survey (54% response rate). All survey respondents (100%) indicated that they participated in the listening sessions due to altruistic motivation, 50% (*n* = 7) had an intellectual motivation, 36% (*n* = 5) were motivated because the request came from a peer or someone they respected, while 29% (*n* = 4) participated for the camaraderie or shared experience.

### 3.2 Key public health data science and leadership concepts for the workforce

Coding methods revealed several concepts under public health data science and public health leadership. Public Health Data Science concepts included data analysis, data management, data interpretation, data visualizations, data-driven decision making, data literacy, surveillance and monitoring, communication with data, data modernization, storytelling with data, data access and sharing, emergent technology, data integration, and data interoperability. Public Health Leadership concepts included policy development, strategic planning, communications, and advocacy, interdisciplinary collaboration and partnership building, crisis management and emergency response, leadership development, health equity and social determinants of health, innovation, systems thinking, and interpersonal skills (Table 4).

**Table 4:** Concepts identified under public health data science and leadership.

Public health data science	Public health leadership
1. Data analysis	1. Policy development
2. Data management	2. Strategic planning
3. Data interpretation	3. Communications and advocacy
4. Data visualizations	4. Interdisciplinary collaboration and partnership building
5. Data-driven decision making	5. Crisis management and emergency response
6. Data literacy	6. Leadership development
7. Surveillance and monitoring	7. Health Equity & social determinants of health
8. Communication with data	8. Innovation
9. Data modernization	9. Systems thinking
10. Storytelling with data	10. Interpersonal skills
11. Data access and sharing	
12. Emergent technology	
13. Data integration	
14. Data interoperability	

The emerging concepts were compatible with resulting concepts from a previous NNPHI public health workforce development project that resulted in a taxonomy used to describe essential skills in the workforce. The framework from that project was applied to clarify and organize the data. Through this application it became clear that data literacy should be recognized as a fundamental concept rather than a subset of data science. As a result, three core concepts emerged: data science, data literacy, and data-informed leadership (15).

#### 3.2.1 Data science

##### 3.2.1.1 Data science defined

While there is variation across sources and organizations regarding definitions of “data science,” all suggest a combination of math and statistics skills often executed using software and/or programming. Data science overlaps with areas like advanced analytics, artificial intelligence, and machine learning.

Work conducted in data science will generally fall into the following process sequence:

- *Data intake:* collecting data from primary or secondary sources and using structured (such as a database of prescription distributions) or unstructured (like focus group transcripts, or online traffic).
- *Data storage*: organizing data so that it can be reliably accessed. Proper storage enables workflow and processes so that the data is usable when and how it needs to be used.
- *Data analytics*: testing and manipulating data using a scientific method. This includes hypothesis testing as well as predictive and generative applications anchored in replicable practices. In this area of work, data visualization can be both process and product as it can help the data scientist assess, but it also is a crucial tool for assisting others who must interpret the data.
- *Data communication*: similar to scientists in other fields, data scientists must use communication skills. The results of their analyses must be presented in a way that empowers administrators to make decisions and community members to act on data results and implications.

##### 3.2.1.2 Data science gaps

Participants from the listening sessions offered many observations related to data science; these included identifying several gaps in the field that impede the workforce. Many public health staff within their departments lacked formal coursework in data science. Additionally, training to support staff in a rapidly changing environment, such as in advances in tools and technology, was absent. Many saw a need to improve the data infrastructure and a need to align academic curriculum with on-the-job needs to better recruit and retain staff. Additionally, participants identified leadership support to provide opportunities for mentorship and career advancement.

In the listening sessions, participants noted that there needed to be a holistic interdisciplinary approach to training/education, to enable current public health students to gain skills like prioritizing practical applications, engaging in data analysis projects, and gaining experience in data management and interpretation. Participants also mentioned a need for formal training in leadership and management; in general, and as it relates to data science, with additional discussion on the challenges of confidently communicating data science results to leaders and by leaders.

The listening sessions also suggested that data science involves high levels of expertise in tool use, i.e., SAS, Excel, machine learning, statistical techniques, experimental methods, and technical writing, all of which were identified as domains in the *Essentials* Framework (15). “Data science practice concepts and skills that must be conveyed to students right out of grad school” a listening session participant noted.

The participants discussed the demand for data-driven courses and curricula for workforce development, noting that current academic curricula did not sufficiently incorporate data science skills. There was a perceived lack of complete and standardized curriculum exclusively focused on data science skills. For example, one participant stated that “teaching them not only how to do the data analysis, but how to sort through what’s data and what’s noise” is a key skill. See Table 5 for a complete listing of “Data Science Gaps” identified by participants.

**Table 5:** Data science gaps identified by participants.

Lack of formal coursework in data science.
Lack of training to support staff in rapidly changing environment (e.g., advances in tools and technology)
Need to improve data infrastructure.
Need to align academic curriculum with on-the-job needs to better recruit and retain staff.
Need for leadership support to provide opportunities for mentorship and career advancement.
Holistic interdisciplinary approach to training/education to gain skills prioritizing practical applications, engaging in data analysis projects, experience in data management and interpretation.
Need for formal training in leadership and management.
Demand for data-driven courses and curricula for workforce development.
Lack of complete and standardized curriculum focused on data science skills.

#### 3.2.2 Data literacy

##### 3.2.2.1 Data literacy defined

Data literacy is the ability to find meaning in data. Just as language literacy is far more than the technical component of letter sounds and grammar, data literacy is the synthesis of a spectrum of knowledge, skills, and attitudes (KSAs) that enable clear communication that retains consistent meaning. Data literacy tasks typically include a combination of the following:

- *Data quality assessment:* knowing when data meets requirements for reliability and appropriate representation of a given phenomenon. When advising leadership, quality assessment helps to know the degree of trust that can be placed in the data analysis results. This includes understanding the appropriateness of certain mathematical and statistical techniques for given data types and objectives.
- *Data logic*: recognizing fallacies and applying logic techniques. Awareness of logical fallacies is a priority for strong data literacy.
- *Data communication*: emphasizing Emotional Intelligence (EI), data literate personnel communicate about data in a manner that can be received, understood, and acted upon. They also recognize the emotional components in data communications, providing resilience against misinformation, bias, or decontextualization.

##### 3.2.2.2 Data literacy gaps

Data literacy, and its required communication and visualization skills, were mentioned as opportunities for training and were part of the most pressing needs of the workforce. Specific opportunities captured include training in data literacy, health literacy, plain language, risk communication, communicating with data, communicating data to leadership for accurate external reporting, data decolonization and community engagement. In addition to noted training opportunities, there is a strong workforce need to understand and communicate data use goals, modernize data systems and visualization, and use systemic behavioral science frameworks to understand issues, quantify health needs and build the general public’s trust.

The listening sessions highlighted the need for alignment of the workforce needs and current public health programs. Participants mentioned they received no academic training in how to communicate data interpretation results to the community, on-the-job training in general risk communication, or public speaking. Data communication skills were not practiced in education but rather learned through professional practice. Participants emphasized the importance of communicating results in plain language to leadership, funders, and stakeholders to help facilitate and ensure the information is subsequently conveyed effectively and accurately by those collaborators. See Table 6 for a complete listing of “Data Literacy Gaps” identified by participants.

**Table 6:** Data literacy gaps identified by participants.

Opportunities to train on data literacy, data communication and visualization skills are pressing needs of the PHW.
Workforce need to understand and communicate data use goals.
Need to modernize data systems and visualizations.
Need to use systemic behavioral science frameworks to understand issues, quantify health needs and build the general public’s trust.
Alignment of workforce needs and current public health programs.
Lack of academic training in how to communicate data interpretation results to the community, on-the-job training in general risk communication and public speaking
Data communication not practiced in education, but rather learned through professional practice.
Need for training in how to communicate results in plain language to leadership, funders, and stakeholders.

#### 3.2.3 Data-informed leadership

##### 3.2.3.1 Data-informed leadership defined

Data-informed Leadership can vary according to what the leadership role itself demands. Generally, Data-informed Leadership uses data to inform, guide, and justify decisions. That said, the leadership role itself (e.g., Team Lead vs. Chief Technical Officer) will dictate the extent to which the following aspects of Data-informed Leadership are included:

- *Data governance*: setting policies that guide data lifecycles. Governance includes standardization and federation of linked information and protocols for handling data at every stage.
- *Data operations:* placing talent, processes, and access to the right tools and products to conduct all data tasking efficiently and effectively while maintaining data security. While this is obviously coupled with data governance, the focus of data operations is on the effective use of data, whereas governance focuses on the appropriate use.
- *Strategic thinking:* determining how data can empower better decision-making outcomes.
- *Data communication:* identifying dissemination policies and practices within and outside the organization.
- *Leadership modeling:* everything that a leader does is an opportunity for emulation (or a cautionary tale against it). Consequently, mindful leadership within Data Operations can model data behaviors such as seeking clarification, requesting additional information, and using data to manage teams.

##### 3.2.3.2 Data-informed leadership gaps

Participants’ responses identify specific leadership-related systems issues gaps that impact the current workforce. These systems issues gaps were discussed within the context of data science leadership, and include the lack of leadership, especially with the loss of institutional knowledge and mentorship when departments experience leadership transitions, the lack of leadership training and data science training as well as the willingness to identify likely candidates for leadership training, and the need for skill-building opportunities in leadership for mid-senior professionals. In terms of leadership capacity, participants revealed a need for comprehensive leadership development programs with a focus on policy development, health equity, crisis management, and data science/ data science communication. They further reported that there is no leadership training and education or explicit path for leadership skills development currently available to them.

Participants also revealed a lack of alignment between current public health programs with data science and leadership curriculum and the workforce preparedness, performance, and practice. Under the leadership curriculum needs, participants listed path for leadership skills, cross-training in analytical tools and process, training in data literacy, health literacy, plain language, risk communication, communicating with data, data decolonization, and community engagement as opportunity areas for continuous professional development. Furthermore, participants identified a need for comprehensive leadership development programs tailored to public health professionals, investment in professional development (at all professional levels), collaboration between academia and public health agencies, and the integration of data science and leadership components into existing curricula. See Table 7 for a complete listing of “Data-informed Leadership Gaps” identified by participants.

**Table 7:** Data-informed leadership gaps identified by participants.

Lack of leadership with the loss of institutional knowledge and mentorship.
Lack of leadership training and data science training.
Lack of willingness to identify likely candidates for leadership training.
Need for skill-building opportunities in leadership for mid-senior professionals.
Need for comprehensive leadership development programs with a focus on policy development, health equity and crisis management
No leadership training and education or explicit path for leadership skills development currently available to them
Systems undermine efforts to create a diver workforce.
Leadership belief that the current system and processes are adequate to create a diverse workforce.
Lack of alignment between current public health programs with data science and leadership curriculum and workforce preparedness, performance, and practice.
Leadership curriculum needs a path for leadership skills, cross training in analytical tools and process, training in data literacy, health literacy, plain language, risk communication, communicating with data, data decolonization, and community engagement.
Need for comprehensive leadership development programs tailored to public health professionals, investment in professional development (at all professional levels), collaboration between academia and public health agencies, and the integration of data science and leadership components into existing curricula.

##### 3.2.3.3 Connecting data science, literacy, and leadership

“Data Science,” “Data Literacy,” and “Data-informed Leadership” are interrelated yet distinct domains. In the conducted listening sessions, participants were encouraged to discuss their most recent engagements with data, which varied across several types:

a) Supervision and review of data analyses conducted by junior personnel, often leading to the provision of feedback or preparation of presentations for stakeholders like organizational leaders or community members.
b) Collection of primary and secondary data, merging technical tasks such as survey design and database management with Emotional Intelligence (EI). Public health workers employ EI to navigate both direct interactions with target populations and interagency communications, accommodating the cultural and organizational norms of all involved entities.
c) Data storytelling, where beyond technical data manipulation, an understanding of data implications is crucial. Participants highlighted the importance of translating data insights into actionable narratives, requiring effective data visualization and robust communication skills.

Given these distinctions, it is vital that workforce development initiatives in public health adequately address the diverse needs and gaps within data science, literacy, and leadership to align with the career goals of public health professionals.

## 4 Discussion

We identified individuals from academia, state, local, and territorial health departments, national public health non-profits, and other related organizations who self-identified having experience in public health leadership, public health workforce development, data science education, public health recruitment and retention, and/or public health leadership curricula. The participant profile is demographically comparable to the United States public health workforce (16).

The listening sessions revealed several key concepts which we organized and simplified into 3 core concepts: data science, data literacy and data-informed leadership. We provided a layman’s definition for each core concept to help bridge the gap in defining public health data science and leadership. These definitions could now be used to address some of the gaps in curriculum development, to guide the preparation and development of the workforce, and to better align practice with academic interpretations.

Within the data science domain, the exploration highlighted the need for technical expertise and the role academia plays. Identified gaps showed that entry-level staff were not ready/familiar with technical software, data analysis, and management principles. There was a disconnect between academia and workforce needs—again, this work presents an opportunity to shrink the gap due to conceptualization of data science and leadership concepts/KSAs.

The listening sessions highlighted the need to better align workforce needs and current public health programs. Participants mentioned they received no academic training in how to communicate data interpretation results to the community, nor did they receive on-the-job training in general risk communication and public speaking. Data communication skills were not practiced in education but rather gained through professional practice. Participants emphasized the importance of communicating results in plain language to leadership, funders, and stakeholders.

For the data literacy domain, there is a pressing need for training in data literacy, communication, and visualization for the workforce. Key areas shown in Table 6 include understanding and communicating data use goals, modernizing data systems and using behavioral science frameworks to address issues, quantify health needs and build public trust. The workforce needs to align with current public health programs but there is a lack of academic training especially in the areas of data interpretation, risk communication, and public speaking. Specific opportunities captured include training in data literacy, health literacy, plain language, risk communication, communicating with data, data decolonization, and community engagement.

Regarding leadership capacity, participants indicated a necessity for extensive leadership development programs emphasizing knowledge and skills policy formulation, health equity, crisis management, and data science/ data science communication. They highlighted a lack of available leadership training and education, noting the absence of a clearly defined pathway for developing leadership competencies. The increasing accessibility of data and the need for collection, analysis, and visualization highlight the disconnect between the public health workforce knowledge and needs, (8) pressuring academic and training institutions to address how to adequately prepare students.

The role of the public health leader has increasingly adapted to a “data-centric” world. In the data-informed leadership domain, there is a values-based approach for public health leaders; this lack also permeates other public health domains, not just data science. The issues exist with most, if not all, leadership competencies being drafted prior to 2018—we did not find one set of public health leadership competencies that included “data literacy, data awareness, data communication” as a competency/skill public health leaders need to have. Data informed public health leaders must not only utilize data and insights to guide their decision-making but also must understand the growing need for more impactful data, and advocate for it. Data modernization efforts must include the development of data-informed leaders.

The listening session information we received about perceived gaps in leadership training and education and lack of clear pathways to leadership capacity was amplified by comments about entry-level staff’s lack of readiness with technical software, data analysis, and data communication, as well as comments suggesting that some existing senior public health leadership was equally lacking competency in the very same data science-related areas. Listening session participants described a challenging dynamic: one that existed between intermediate-level staff trying to effectively communicate public health data science results to leadership, and leadership’s difficulty understanding those results. Additionally, there was the perception by some that senior leadership would incorrectly transfer the information upstream or selectively communicate the information to external audiences such as government officials, funders, the media, and the public. Whether these perceptions are limited to the listening session participants or can be generalized to the larger workforce, they highlight the need for additional training and education in public health data science for everyone who engages with public health data and data communication within their public health roles, regardless of agency division or rank.

Based on the listening session content analysis, we determined that further research was warranted to explore data science and leadership in the public health workforce. The results of that research are not included in this paper but revealed the importance of integrating competencies in data literacy and data communication into other public health leadership competencies and paving a pathway of *public health data science leadership*. Such an integration and pathway would not require a significant departure from either subject; a leader in general public health and a leader in public health data science have many traits in common: both must have as a motivating force a desire to affect positive change and transform the status quo; both must know how to develop a vision with and for their teams, programs, and agencies, as well as the knowledge, skills, and abilities (KSAs) to guide others on the path to reach those long-term goals and objectives. These common KSA’s include, but are not limited to, establishing credibility through both the development of trust and technical expertise in their fields and achieving competency in how to effectively communicate in a way that information is transmitted but, more importantly, understood and acted upon. As we explored the many facets of the intersection between public health data science and public health leadership, we ultimately reached the understanding and agreement that there is no public health leadership competency without public health data science competency; data is the foundation of the core functions of public health, and without competency in at least the basic KSA’s of its application and communication, there can be no comprehensive competency in public health leadership.

We further suggest that there are two crucial areas where this integration of public health data science competency into public health leadership competency can take place. The first, at the academic level through curriculum that has been developed in a collaborative effort between academicians and practitioners. This includes academicians helping practitioners understand the academic process and content that must be considered in the development of collaborative curriculum, such as a primary focus on theory and the key relationships between curriculum and accreditation. This equally includes practitioners helping academicians understand the elements of curriculum that must be considered for practice, such as hands-on practical experience and meaningful goal and competency-focused field experiences. A second method for the integration of data science competencies into public health leadership competencies is through standardized mentor programs within the practice agencies; mentorships that include public health data science and leadership training which can then be conveyed in a standardized format to mentees. A program such as this certainly requires resources and prioritization within the already stretched capacity of public health practice agencies, yet that prioritization is ultimately the key to successful integration—the field of public health must prioritize competency in both public health data science and in public health leadership. Without that prioritization competency will not exist in either discipline at the level required in the current and future public health landscape.

While the participant population reflects the US public health workforce at large, it is limited by the lack of diversity of the respondents and has potential for bias with the self-declaration of experience in the topic areas. To ensure inclusivity and diversity, demographic distributions were continuously assessed and focused efforts were made to reach out to specific groups which were underrepresented in responses received. Additionally, selection bias may have occurred based on network proximity. To mitigate selection bias, only 1 evaluation team member was involved in the process of selecting LS participants based on the self-reported experience recruitment question.

Limitations inherent in qualitative methods were controlled by utilizing a mixed methods approach which included the use of theoretically informed processes in data collection and analysis as well as the use of three independent coders to identify themes and categories.

Our results also aligned with research in the field that highlights the lack of investment in foundational public health services. This lack of financial support continues to contribute to the challenges in better equipping the workforce with the skills in data science and leadership required in public health practice.

## 5 Conclusion

The work presented herein provides a foundational step toward strengthening the public health workforce by identifying key concepts in public health data science and leadership, as well as pinpointing gaps in public health data science and leadership capacity, accessibility, training, and educational needs. Listening sessions were conducted, and participants shared their experiences with data science and leadership. These discussions led to the classification of three fundamental concepts: data science, data literacy, and data-informed leadership. Clear, layman-friendly definitions of these concepts have been developed, aimed at addressing gaps in curriculum development and data modernization efforts, and they serve as essential tools for advocating for sustained resource investment in public health data science and leadership domains.

## Data Availability

The raw data supporting the conclusions of this article will be made available by the authors, without undue reservation.
