# Supplementary material for: Bridging the gap: enhancing data science and leadership knowledge and skills in the context of the public health workforce
Source: Front Public Health. 2025 Apr 29;13:1505869. doi: 10.3389/fpubh.2025.1505869 (PMC12076155; doi:10.3389/fpubh.2025.1505869)
Supplement: Supplementary file 1 [file Table_1.docx]

Supplemental Table 1: Participant Profile

| **Table 1: Participant Profile** | **Recruitment Survey (N=65) %** | | **Listening Session (N=26) %** | |
| --- | --- | --- | --- | --- |
| **Q3 Degrees (Multiple Selections)** |  |  |  |  |
| Some high school | 2 | 3% | 0 | 0% |
| High school degree or equivalent (e.g., GED) | 32 | 49% | 9 | 35% |
| High school technical | 0 | 0% | 0 | 0% |
| Some college but no degree | 0 | 0% | 0 | 0% |
| Associate degree or certificate | 3 | 5% | 2 | 8% |
| Bachelor’s degree | 36 | 55% | 16 | 62% |
| Master’s degree | 48 | 74% | 20 | 77% |
| Doctoral degree (MD, DO, PhD, DDS, JD, etc.) | 18 | 28% | 12 | 46% |
| Other | 0 | 0% | 0 | 0% |
| **Q4 Race-Ethnicity (Multiple Selections)** |  |  |  |  |
| Asian | 14 | 22% | 4 | 15% |
| American Indian or Alaskan Native | 0 | 0% | 0 | 0% |
| Black/African American | 7 | 11% | 3 | 12% |
| Native Hawaiian or other Pacific Islander | 1 | 2% | 0 | 0% |
| Hispanic, Latino or Spanish origin of any race | 6 | 9% | 4 | 15% |
| Non-Hispanic | 5 | 8% | 2 | 8% |
| White | 36 | 55% | 15 | 58% |
| Two or more | 3 | 5% | 1 | 4% |
| Some other race | 1 | 2% | 0 | 0% |
| Decline to state | 3 | 5% | 1 | 4% |
| **Q6 Gender** |  |  |  |  |
| Male | 19 | 29% | 5 | 19% |
| Female | 46 | 71% | 21 | 81% |
| Non-Binary | 0 | 0% | 0 | 0% |
| Prefer not to say | 0 | 0% | 0 | 0% |
| Other | 0 | 0% | 0 | 0% |
| **Q7 Expertise** |  |  |  |  |
| Public health leadership | 41 | 63% | 18 | 69% |
| Public health workforce development | 38 | 58% | 19 | 73% |
| Public health programs with data science | 55 | 85% | 20 | 77% |
| Public health recruitment and retention | 24 | 37% | 11 | 42% |
| Public health leadership curricula | 14 | 22% | 9 | 35% |
| No, I do not have experience in any of these areas. | 4 | 6% | 0 | 0% |
| **Q8 Years of Experience** |  |  |  |  |
| Less than 6 months | 2 | 3% | 0 | 0% |
| 6 months to 1 year | 3 | 5% | 0 | 0% |
| 2 years to 5 years | 21 | 32% | 9 | 35% |
| 6 years to 10 years | 19 | 29% | 9 | 35% |
| 11 years to 15 years | 10 | 15% | 5 | 19% |
| Greater than 15 years | 9 | 14% | 3 | 12% |
| No Response | 1 | 2% | 0 | 0% |
| **Q9 Supervision** |  |  |  |  |
| Yes | 49 | 75% | 23 | 88% |
| No | 15 | 23% | 3 | 12% |
| No Response | 1 | 2% | 0 | 0% |
| **Q9a Years of Supervision/Management** |  |  |  |  |
| Less than 6 months | 3 | 5% | 1 | 4% |
| 6 months to 1 year | 3 | 5% | 1 | 4% |
| 2 years to 5 years | 24 | 37% | 13 | 50% |
| 6 years to 10 years | 13 | 20% | 6 | 23% |
| 11 years to 15 years | 1 | 2% | 1 | 4% |
| Greater than 15 years | 5 | 8% | 1 | 4% |
| **Q10 Current Work Setting** |  |  |  |  |
| Local health agency | 16 | 25% | 7 | 27% |
| State health agency | 14 | 22% | 5 | 19% |
| Territorial health agency | 0 | 0% | 0 | 0% |
| Federal health agency | 2 | 3% | 0 | 0% |
| Tribal health agency | 0 | 0% | 0 | 0% |
| Educational/academic institution | 12 | 18% | 7 | 27% |
| Private nonprofit organization | 10 | 15% | 5 | 19% |
| Private for-profit organization | 5 | 8% | 0 | 0% |
| Personal health service industry | 0 | 0% | 0 | 0% |
| Other | 5 | 8% | 2 | 8% |
| No Response | 1 | 2% | 0 | 0% |
| **Q11 Employer where developed experience** |  |  |  |  |
| Local health agency | 16 | 25% | 8 | 31% |
| State health agency | 19 | 29% | 7 | 27% |
| Territorial health agency | 0 | 0% | 0 | 0% |
| Federal health agency | 4 | 6% | 1 | 4% |
| Tribal health agency | 0 | 0% | 0 | 0% |
| Educational/academic institution | 11 | 17% | 5 | 19% |
| Private nonprofit organization | 8 | 12% | 5 | 19% |
| Private for-profit organization | 3 | 5% | 0 | 0% |
| Personal health service industry | 0 | 0% | 0 | 0% |
| Other | 3 | 5% | 0 | 0% |
| No Response | 1 | 2% | 0 | 0% |
| **Q12 General Work Area** |  |  |  |  |
| Urban | 30 | 46% | 10 | 38% |
| Suburban | 18 | 28% | 7 | 27% |
| Rural | 3 | 5% | 2 | 8% |
| Tribal area | 1 | 2% | 0 | 0% |
| Other | 12 | 18% | 7 | 27% |
| No Response | 1 | 2% | 0 | 0% |
| **Q13 Occupation** |  |  |  |  |
| Academia- Curriculum developers, Professors/Faculty, Coordinators/Staff | 7 | 11% | 3 | 12% |
| Academia- Current students from public health programs | 3 | 5% | 2 | 8% |
| State, local, tribal, and territorial health departments, and federal agencies- Programmatic Staff | 17 | 26% | 6 | 23% |
| State, local, tribal, and territorial health departments, and federal agencies- Directors & Managers | 16 | 25% | 7 | 27% |
| State, local, tribal, and territorial health departments, and federal agencies- Administrative staff | 2 | 3% | 1 | 4% |
| State, local, tribal, and territorial health departments, and federal agencies- Recent graduates | 2 | 3% | 0 | 0% |
| National public health non-profits and other related organizations – Programmatic Staff | 3 | 5% | 2 | 8% |
| National public health non-profits and other related organizations – Directors & Managers | 7 | 11% | 5 | 19% |
| National public health non-profits and other related organizations –Administrative staff. | 2 | 3% | 0 | 0% |
| National public health non-profits and other related organizations –Recent Graduates | 0 | 0% | 0 | 0% |
| Private-for-profits and other related organizations-Programmatic Staff | 1 | 2% | 0 | 0% |
| Private-for-profits and other related organizations- Direct and Managers | 0 | 0% | 0 | 0% |
| Private-for-profits and other related organizations- Recent graduates | 1 | 2% | 0 | 0% |
| Other (e.g., volunteer, intern) | 2 | 3% | 0 | 0% |
| No Response | 2 | 3% | 0 | 0% |
